# Developmental changes in attention to social information from childhood to adolescence in autism spectrum disorders: a comparative study

**DOI:** 10.1186/s13229-020-00321-w

**Published:** 2020-04-09

**Authors:** Toru Fujioka, Kenji J. Tsuchiya, Manabu Saito, Yoshiyuki Hirano, Muneaki Matsuo, Mitsuru Kikuchi, Yoshihiro Maegaki, Damee Choi, Sumi Kato, Tokiko Yoshida, Yuko Yoshimura, Sawako Ooba, Yoshifumi Mizuno, Shinichiro Takiguchi, Hideo Matsuzaki, Akemi Tomoda, Katsuyuki Shudo, Masaru Ninomiya, Taiichi Katayama, Hirotaka Kosaka

**Affiliations:** 1 Department of Science of Human Development, Humanities and Social Science, Faculty of Education, University of Fukui, Fukui, Fukui Japan; 2 Research Center for Child Mental Development, University of Fukui, Eiheiji, Fukui, Japan; 3 Department of Child Development, United Graduate School of Child Development, Osaka University, Kanazawa University, Hamamatsu University School of Medicine, Chiba University, and University of Fukui, Suita, Osaka, Japan; 4 Research Center for Child Mental Development, Hamamatsu University School of Medicine, Hamamatsu, Shizuoka, Japan; 5 Department of Neuropsychiatry, Graduate School of Medicine, Hirosaki University, Hirosaki, Aomori, Japan; 6 Research Center for Child Mental Development, Chiba University, Chiba, Chiba Japan; 7 Department of Pediatrics, Faculty of Medicine, Saga University, Saga, Saga Japan; 8 Research Center for Child Mental Development, Kanazawa University, Kanazawa, Ishikawa Japan; 9 Division of Child Neurology, Department of Brain and Neurosciences, Faculty of Medicine, Tottori University, Yonago, Tottori, Japan; 10 Institute of Human and Social Sciences, Kanazawa University, Kanazawa, Ishikawa Japan; 11 Tottori University Hospital, Yonago, Tottori, Japan; 12 Department of Child and Adolescent Psychological Medicine, University of Fukui Hospital, Eiheiji, Fukui, Japan; 13 Development Center, Healthcare Business Division, JVCKENWOOD Corporation, Yokohama, Kanagawa Japan; 14 Department of Neuropsychiatry, Faculty of Medical Sciences, University of Fukui, Eiheiji, Fukui, Japan

**Keywords:** Autism spectrum disorder, Social information, Eye-tracking, Developmental change

## Abstract

**Background:**

Elucidating developmental changes in the symptoms of autism spectrum disorder (ASD) is important to support individuals with ASD. However, no report has clarified the developmental changes in attention to social information for a broad age range. The aim of this study was to investigate the developmental changes in attention to social information from early childhood to adolescence in individuals with ASD and typically developed (TD) children.

**Methods:**

We recruited children with ASD (*n* = 83) and TD participants (*n* = 307) between 2 and 18 years of age. Using the all-in-one-eye-tracking system, Gazefinder, we measured the percentage fixation time allocated to areas of interest (AoIs) depicted in movies (the eyes and mouth in movies of a human face with/without mouth motion, upright and inverted biological motion in movies showing these stimuli simultaneously, people and geometry in preference paradigm movies showing these stimuli simultaneously, and objects with/without finger-pointing in a movie showing a woman pointing toward an object). We conducted a three-way analysis of variance, 2 (diagnosis: ASD and TD) by 2 (sex: male and female) by 3 (age group: 0–5, 6–11, and 12–18 years) and locally weighted the scatterplot smoothing (LOESS) regression curve on each AoI.

**Results:**

In the face stimuli, the percentage fixation time to the eye region for the TD group increased with age, whereas the one for the ASD group did not. In the ASD group, the LOESS curves of the gaze ratios at the eye region increased up to approximately 10 years of age and thereafter tended to decrease. For the percentage fixation time to the people region in the preference paradigm, the ASD group gazed more briefly at people than did the TD group.

**Limitations:**

It is possible that due to the cross-sectional design, the degree of severity and of social interest might have differed according to the subjects’ age.

**Conclusions:**

There may be qualitative differences in abnormal eye contact in ASD between individuals in early childhood and those older than 10 years.

## Background

Autism spectrum disorder (ASD) is a neurodevelopmental disorder characterized by a “deficit of social communication and social interaction” and “restricted, repetitive patterns of behavior, interests, or activities,” comprising several specific symptoms [1]. The core features of ASD persist throughout life. However, it is considered that the clinical presentation of ASD may change during development [2, 3]. It would be beneficial for diagnosis, treatment, and support to clarify changes in the clinical picture of ASD associated with the developmental stages.

Most, but not all, previous studies have reported that individuals with ASD gaze less at social information regardless of age. For example, the face, especially the eye and mouth regions, conveys salient social information, and it is believed that people prefer to pay attention to these areas from infancy [4]. When shown videos and photographs in which people appear, children with ASD [5–12] and adults with ASD [13] are both reported to gaze at the human faces for shorter periods of time than do typically developed (TD) individuals. Studies with various types of stimuli have reported that, of the parts of the face, the region around the eyes is particularly looked closely at more briefly, by both children with ASD [9, 12, 14–20] and adults with ASD [11, 18, 21–25]. When the TD and ASD groups were simultaneously shown movies of people and geometric shapes, i.e., the preference paradigm, which was created under the hypothesis that individuals with ASD prefer to pay attention to highly repetitive images than to social images [26], the ASD group observed people for shorter periods of time than did the TD group. This gaze behavior has been reported in both children with ASD [26–30] and adults with ASD [22]. Moreover, when TD children and children with ASD were shown movies of biological motion, in which multiple points moved in concert and created movements that resemble those of living beings, children with ASD were reported to gaze at biological motion for shorter periods than did the TD children [31–33]. It is considered that the preference for biological motion is a fundamental mechanism facilitating adaptive interaction with other living beings [31] and that the lesser attention to biological motion in the ASD groups appears to be more specific to the processing of biological motion than to a purely perceptual problem [34]. In a video of joint attention that used finger-pointing movements, recognized as an equally attractive social stimulus for humans as that described above [4], children with ASD reportedly reacted less accurately to finger-pointing than did the TD children [35] or showed a lower ratio of gazing at an object to which a finger had been pointed [36]. However, when presented only with people’s faces on a monitor in a passive view condition, children in an ASD group showed comparable attention to the eye region with those in a TD group [5, 37], while post-pubescent participants in an ASD group showed less attention to the eye region than did post-pubescent participants in a TD group [14, 22]. Group differences between ASD and TD were more likely to occur with dynamic stimuli containing several individuals than with dynamic stimuli containing a single individual [16]. Moreover, group differences between ASD and TD in attention to social information did not occur on biological motion in adults [22], on the preference paradigm when the stimuli contained only one individual in children [27], and on cartoon-like images with a human figure in school-aged children [38]. Thus, although individuals with ASD tend not to pay attention to social information, this appears to ultimately depend on the quality of the stimulus and the participant’s age.

This gazing at social information reportedly changes during development, and the developmental trajectories between ASD and TD groups appear to differ. For example, when a scene from a TV program or a photograph of a person’s face was shown to both TD adults/children and adults/children with ASD, TD children tended to gaze more briefly at the eye region than did TD adults; however, it was reported that no significant differences between children and adults with ASD were observed in the gaze ratio at the eyes and mouth regions [11, 18]. In addition, a study presented a video of a face, the preference paradigm, biological motion, and finger-pointing to TD children aged 4.0 ± 1.9 years and children with ASD aged 4.8 ± 1.1 years [37]. In the preference paradigm, the gaze ratio at the people region decreased and that of the geometric shapes increased with age in both the ASD and TD groups. In the TD group, the gaze ratio at the region around the mouth in a video of a face and the ratio of gazing at the non-object being pointed at in the movie of finger-pointing increased with age. In addition, the ratio of gazing at the object being pointed at in the movie of finger-pointing decreased with age in the TD group. Conversely, the participants in the ASD group did not show other significant correlations. Although a weakness of that study was that the sample size was small, the results showed that participants with ASD did not show the developmental changes in attention to social information that TD participants showed in childhood [37]. To summarize, depending on the type of stimulus, participants in the ASD group appeared not to show the maturation of the fixation pattern for faces that occurs from childhood to adulthood in TD individuals.

However, no study targeting either TD individuals or individuals with ASD has thus far identified the developmental changes in gazing at social information targeting subjects covering a broad age range after childhood. In addition, there is a discrepancy, especially in TD, in that although gazing at areas carrying strong social information decreases or does not change along with increasing age in childhood, adults often gaze longer at such information than do children. Identifying the developmental changes in gazing at social information, targeting a broad range of age groups after childhood, will help resolve this contradiction. It is also assumed that developmental changes in gazing at social information depend on the quality of the stimulation; hence, identifying the quality of stimulation that the subjects react to sensitively would offer suggestions for elucidating the mechanism by which social difficulties occur in children and adults with ASD. In this study, therefore, we aimed at identifying the developmental changes in gazing at social information in individuals with ASD and TD, from childhood to adolescence. We used Gazefinder (JVC KENWOOD Corporation, Kanagawa, Japan), an eye-tracking device that can measure gazing at social information with multiple stimuli of different qualities.

## Methods

### Participants

#### The ASD group

A total of 108 children between 3 and 17 years old (18 boys and 8 girls in the 0–5-year group, 37 boys and 10 girls in the 6–11-year group, and 25 boys and 10 girls in the 12–18-year group) with ASD were recruited from the University of Fukui Hospital, Hamamatsu University Hospital, Hirosaki University Hospital, Chiba University Hospital, Kanazawa University Hospital, Tottori University, Saga University Hospital, and Osaka University Hospital, Japan. All participants were of Japanese ethnicity. The participants were diagnosed with ASD or any other psychopathological condition by a certified psychiatrist of the Japanese Board of Psychiatry or board-certified pediatrician of the Japan Pediatric Society, based on the criteria of the Diagnostic and Statistical Manual of Mental Disorders, fifth edition [1]. Although we permitted attention deficit hyperactivity disorder as a comorbid disorder, we excluded participants that met the diagnostic criteria for any other psychopathological condition. The IQ of all participants with ASD was assessed with the Wechsler Intelligence Scale for Children-Fourth Edition (WISC-IV), or the Tanaka-Binet Test (Japanese version of the Stanford-Binet Test), or the developmental quotient (DQ) of the Kyoto Scale of Psychological Development. All participants had an IQ/DQ of 70 or higher.

#### TD group

We also recruited 374 TD participants between 2 and 18 years old (81 boys and 78 girls in the 0–5-year group, 80 boys and 89 girls in the 6–11-year group, and 20 boys and 26 girls in the 12–18-year group) from the local community. All participants were of Japanese ethnicity. On the face sheet of the questionnaire, we confirmed that for the TD participants, there was no indication of disorder at the medical checkups for 1.5-year-old and 3-year-old children conducted by pediatricians and public health nurses in Japan, and that they had not been diagnosed with mental disorders and had no active diseases requiring continuous hospital visits.

#### Stimuli

We utilized Gazefinder, an all-in-one eye-tracking system where hardware and stimulating videos are grouped together to evaluate the percentages of fixation time allocated to specific objects (see below) on a video monitor. Participant eye positions were measured using infrared light sources and cameras located below a 19-inch thin-film transistor (1280 × 1024 pixels). Using corneal reflection techniques, eye position was recorded as (X, Y) coordinates at a frequency of 50 Hz (i.e., 3000 data collections/min). The calibration of eye position recordings was performed using a five-point method. It is recommended to retain the distance between the face and the monitor at approximately 70 cm.

**Fig. 1:**
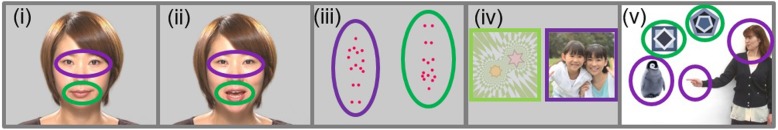
Gazefinder movie samples and their areas of interest (AoIs). (i) Screenshot of the human face without mouth motion; AoI-1 and AoI-2 include the eye and mouth regions, respectively; (ii) Screenshot of the human face with mouth motion; AoI-1 and AoI-2 include the eye and mouth regions, respectively; (iii) Screenshot of biological motion; AoI-1 and AoI-2 are the upright and inverted images, respectively; (iv) Screenshot of the preference paradigm; AoI-1 and AoI-2 are people and geometry, respectively; (v) Screenshot of finger pointing; AoI-1 and AoI-2 are social and geometry areas, respectively

After calibration of the eye position with a five-point method, Gazefinder presented five types of movies; (A) human faces without mouth motion, (B) human faces with mouth motion, (C) biological motion of a human, (D) the preference paradigm, and (E) finger-pointing. (A) Human faces without mouth motion included movies of a still face (4 s), of the eyes blinking (an actress repeatedly opens and closes her eyes for 5 s), and of a still face (an actress with a still face appears for 5 s, and this movie was presented after the mouth moving face movie described below). (B) Human faces with mouth motion included movies of a mouth moving face (an actress repeatedly opens and closes her mouth for 5 s) and of a talking face (7 s). In the talking face movie, the actress says, “Konnichiwa” (“Hello”), “Onamaewa?” (“What is your name?”), and “Issyoniasobouyo” (“Let’s play together”). Face stimuli are considered representative of social stimuli [4], and humans appear to naturally pay attention to the face, especially to the eye area [39]. The difference between “(A) human faces without mouth motion” and “(B) human faces with mouth motion” is that the moving mouth interferes with directing attention to the eye region. Moreover, previous studies have reported that attention to the mouth region increases when individuals are shown movies of talking faces or of faces with the mouth in motion [22, 39]. (C) Biological motion movies presented upright and inverted biological motion simultaneously for 11 s. The movie was accompanied by the song “Under the Big Chestnut Tree” to which an upright human danced. The biological motion movie measures the degree of attention to social information under the hypothesis that humans show an innate preference for a biological motion to facilitate adaptive interaction with other living beings [31]. In addition, a brain imaging study with adults suggested that the activated brain areas processing inverted biological motion differed from those processing upright biological motion [40]. (D) The preference paradigm movies simultaneously showed people and geometric shapes at the same size (20 s) and geometric shapes in small-frame images in a small window embedded in movies of people (16 s). The preference paradigm movie measures the degree of attention to social information under the hypothesis that individuals with ASD prefer to pay attention to highly repetitive images rather than social images [26]. (E) Finger-pointing movies presented objects with or without finger-pointing (8 s). Finger-pointing, also known as joint attention, concerns a shared attention state between two individuals focused on an object/event of interest [41] and is categorized as representative of social stimuli [4], as described above. Therefore, it is suggested that the degree of attention to social information can be determined by measuring the response to these types of stimuli. Each stimulus was presented once, and the presentation order of these stimuli was randomly predetermined; all participants saw the stimuli in the same order. A music box sound was played while presenting the stimuli except for (C) biological motion. Figure 1 presents samples of the stimuli.

Percentage fixation times allocated to areas of interest (AoIs) on the video monitor were automatically calculated (time allocated to a particular area/duration of stimulus presentation). The stimulus movies were loaded on Gazefinder, and the AoIs of each stimulus were also set by default; therefore, there was no need for the experimenter to change the setting to derive the percentage fixation times allocated to the AoIs. The AoIs in each stimulus are presented in Fig. 1.

#### Procedures

The study protocol was approved by the ethics committee of each university and conformed to the tenets of the Declaration of Helsinki (as revised in 2000). After a complete explanation of the study, all participants or their parents/legal guardians provided written informed consent. Participants completed the Gazefinder test in a quiet room in each facility. For the ASD group, IQ/DQ evaluation was performed if it had not taken place within the past 2 years. The parents/legal guardians of the participants completed the Social Responsiveness Scale Second Edition (SRS-2), which is a questionnaire used to determine the severity of social deficits [42]. The SRS-2 consists of 65 items, and total SRS scores range from 0 to 195, with higher total scores indicating more severe social deficits. There are SRS-2 forms for preschool children (2.5–4.5 years), school-age children (4-18 years), and adults (ages 19 and up). We used the SRS-2 preschool form for participants aged 2 to 3 years and the SRS-2 school-age form for participants older than 4 years.

#### Analysis of data

Exclusion criteria

As a reference to the Japanese cutoff score of the SRS-2, the cutoff score for the SRS-2 preschool form was 48.5 (sensitivity, 0.83; specificity, 0.82) [43], and the cutoff scores for the SRS-2 school-age form were 53.5 for boys (sensitivity, 0.91; specificity, 0.48) and 52.5 for girls (sensitivity, 0.89; specificity, 0.41) [44]; we excluded participants with ASD who were below and TD participants who were above the cutoff point. Additionally, individuals were excluded when their available percentage of fixation time was < 70% (i.e., Gazefinder could not detect the eye position more than 30% of the stimulus presentation time).

#### Statistical analysis

All statistical analyses were conducted using IBM SPSS, version 24 (IBM Corp., Armonk, NY). At first, for age comparisons, we carried out two-way analysis of variance (ANOVA), 2 (diagnosis: ASD and TD) by 2 (sex: male and female), for each age group (age groups: 0–5-year group, 6–11-year group, and 12–18-year group). We set .05 as the significance level. Second, we conducted a three-way ANOVA for the SRS-2 total score, 2 (diagnosis) by 2 (sex) by 3 (age group). We set .05 as the significance level. Third, we conducted a three-way ANOVA on each AoI, 2 (diagnosis) by 2 (sex) by 3 (age group), for clarifying the effects in gaze patterns of these factors. To avoid type 1 statistical errors, we applied Bonferroni corrections and set .05 divided by the total number of AoIs as the significance level; thus, we set *p* = .005 (.05/10) as the significance level. We also applied Bonferroni corrections for post hoc comparisons. However, we could only grasp rough developmental changes in gazing at social information using this method. In addition, as described above, developmental changes in gazing at social information were unknown. Therefore, we finally used locally weighted scatterplot smoothing (LOESS) [45, 46] to graphically evaluate the continuous effect of age on attention to social information. LOESS is a procedure for fitting a regression surface to data through multivariate smoothing [46]. We used the LOESS kernel Epanechnikov method and set the % of points to fit to 50%, which is the default value of LOESS analysis in IBM SPSS version 24. We used the kernel Epanechnikov method, which is one of the most popular, for pattern analysis [47]. Finally, with consideration to the analysis results described above, we carried out a correlation analysis between the SRS-2 scores and the percentage fixation time to AoIs and correlation analysis between the IQ/DQ scores and the percentage fixation time to AoIs. As IQ/DQ was only measured in the ASD group, the latter is only for the ASD group. We set *p* = .005 as the significance level following the rationale used for the previous analyses.

## Results

Applying the exclusion criteria described above, there were 83 participants in the ASD group (63 boys and 20 girls): 19 (13 boys and 6 girls) in the 0–5-year group, 34 (28 boys and 6 girls) in the 6–11-year group, and 30 (22 boys and 8 girls) in the 12–18-year group. In the TD group, the final participants were 307 (141 boys and 166 girls); the 0–5-year group included 120 (58 boys and 62 girls) participants, the 6–11-year group, 150 (69 boys and 81 girls) participants; and the 12–18-year group, 37 (14 boys and 23 girls) participants. The characteristics of the participants included in the analysis are summarized in Table 1.

**Table 1:** Characteristics of the participants

		*n*	Age	IQ/DQ	SRS-2	Comorbid ADHD
ASD						
0–5-year group	**Total**	**19**	**4.7 ± 0.9**	**89.1 ± 10.3**	**72.8 ± 17.3**	**10**
	Male	14	4.6 ± 1.0	89.1 ± 9.3	70.0 ± 19.0	8
	Female	5	5.0 ± 0.5	89.2 ± 13.9	80.6 ± 8.7	2
6–11-year group	**Total**	**34**	**9.2 ± 1.6**	**94.6 ± 14.6**	**88.5 ± 25.6**	**17**
	Male	28	9.0 ± 1.6	95.0 ± 15.1	88.3 ± 26.7	15
	Female	6	10.0 ± 1.6	92.7 ± 13.0	89.3 ± 21.2	2
12–18-year group	**Total**	**30**	**14.5 ± 1.6**	**97.3 ± 14.6**	**88.3 ± 24.7**	**14**
	Male	22	14.4 ± 1.7	101.0 ± 13.7	88.7 ± 23.1	11
	Female	8	14.9 ± 1.4	87.3 ± 12.8	87.3 ± 30.5	3
TD						
0–5-year group	**Total**	**120**	**4.6 ± 1.0**	–	**29.6 ± 11.8**	**–**
	Male	58	4.5 ± 1.0	–	31.3 ± 10.7	**–**
	Female	62	4.7 ± 1.0	–	28.0 ± 12.6	**–**
6–11-year group	**Total**	**150**	**8.7 ± 1.7**	–	**23.8 ± 11.8**	**–**
	Male	69	8.6 ± 1.6	–	24.4 ± 11.8	**–**
	Female	81	8.8 ± 1.7	–	23.3 ± 11.9	**–**
12–18-year group	**Total**	**37**	**14.2 ± 1.5**	–	**21.5 ± 11.7**	**–**
	Male	14	13.2 ± 0.8	–	19.7 ± 10.3	**–**
	Female	23	14.8 ± 1.6	–	22.5 ± 12.6	**–**

For age, in the 6–11-year group, there was a significant main effect of age group (*F* (1, 180) = 4.29, *p* = .040, *η*^2^ = 0.023), with the participants in the ASD group being significantly older than those in the TD group. In the 12–18-year group, there was a significant main effect of sex (*F* (1, 63) = 7.29, *p* = .009, *η*^2^ = 0.104), with female participants being significantly older than male participants. Other main effects and interactions were not significant. For SRS-2, there was a significant main effect of diagnosis (*F* (1, 378) = 673.76, *p* < .001, *η*^2^ = 0.641), and a diagnosis × age group interaction (*F* (2, 378) = 8.35, *p* < .001, *η*^2^ = 0.042). Post hoc comparisons applying Bonferroni corrections revealed that the SRS-2 total score in the 0–5-year group was significantly higher than those in the other age groups in TD group and lower than those in the other age groups in ASD group.

### Face without mouth motion

For the eye region, there was a significant main effect of age group (*F* (2, 378) = 6.68, *p* = .001, *η*^2^ = 0.034) and none of the other main effects and interactions were significant (Fs ≤ 5.20, ps ≥ .023). Post hoc comparisons, applying Bonferroni corrections, revealed that the percentage fixation times in the 12–18-year group were significantly higher than those in the other groups (ps < .001), and the percentage fixation times in the 6–11-year group were significantly higher than those in the 0–5-year group (*p* < .001). Based on the LOESS plot, the ASD group showed a sharp decline of attention to the eye region after around 10 years of age, while there was a constant rise of attention to the eye region after around 5 years of age in the TD group.

For the mouth region, the diagnosis × age group interaction was significant (*F* (2, 378) = 5.80, *p* = .003, *η*^2^ = 0.030), and the percentage fixation times in the 12–18-year group were significantly lower than those in the 0–5-year and 6–11-year groups in the TD group (*p* < .001). There were no significant differences among the age groups in the ASD group (*p* > .10). The LOESS plot showed that there was a sharp increase of attention to the mouth region in the ASD group after around 10 years of age, whereas there was a constant decline of attention to the mouth region in the TD group after around 5 years of age.

**Fig. 2:**
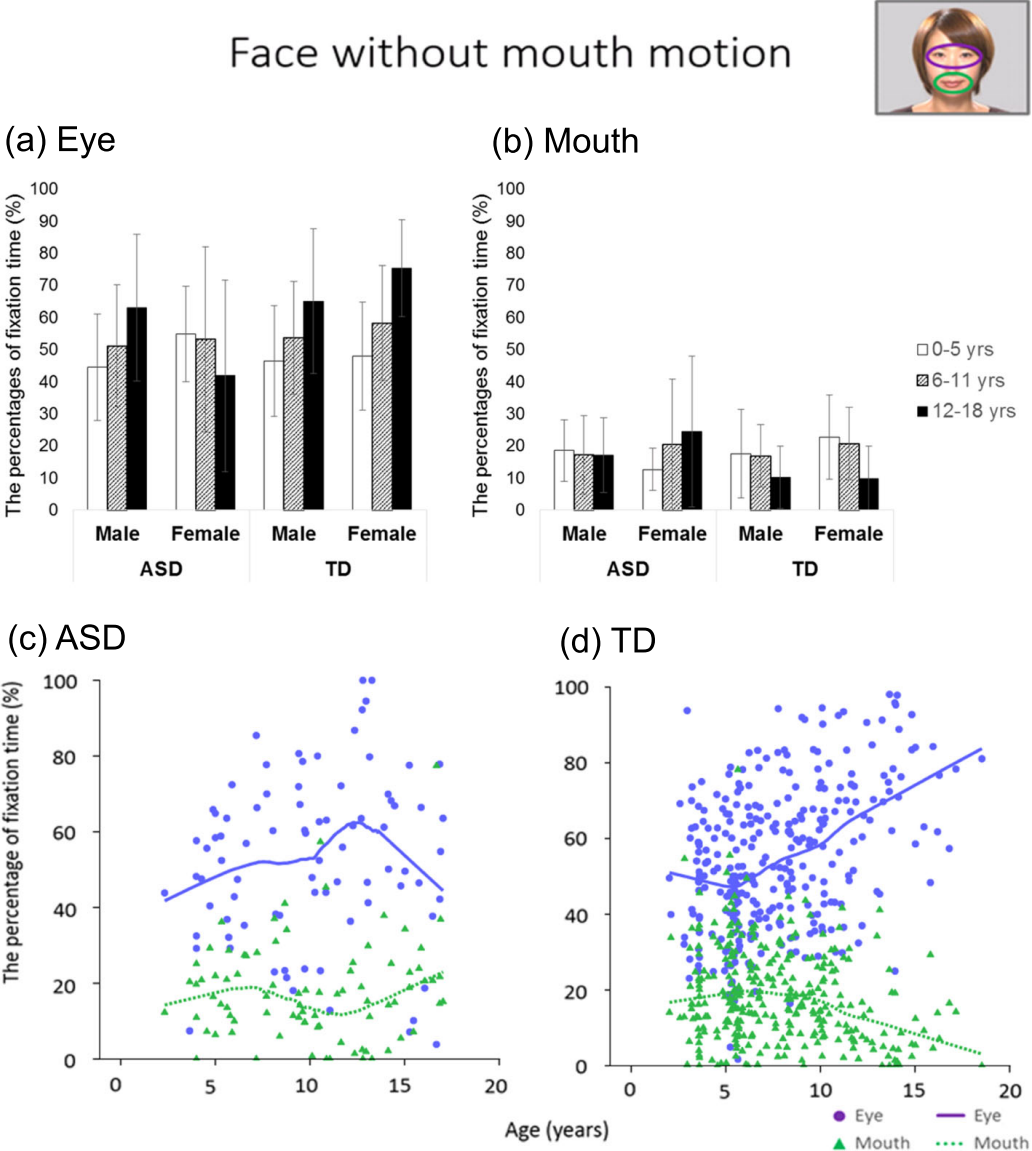
Bar graphs and LOESS curves for “face without mouth motion.” **a**, **b** The bar graphs of the ASD group and TD group for the percentage fixation times of the eye and mouth regions, respectively. Error bars indicate standard errors of the mean. **c**, **d** Scatter plots and LOESS curves of the ASD group and TD group, respectively. Purple circles and lines show data from the eye region, and yellow green triangles and dashed lines show data from the mouth region

These results are shown in Table 2 and Fig. 2

**Table 2:** Results of the ANOVA

	Diagnosis		Sex		Age group		Diagnosis × sex		Diagnosis × age group		Sex × age group		Diagnosis × sex × age group	
	*F*	*P*	*F*	*P*	*F*	*P*	*F*	*P*	*F*	*P*	*F*	*P*	*F*	*P*
Face without mouth motion														
Eye	5.20	.023	0.20	.652	***6.68***	.001*	2.30	.130	4.36	.013	1.46	.233	4.41	.013
Mouth	1.33	.249	1.41	.235	1.26	.286	0.11	.745	***5.80***	.003*	0.47	.628	2.05	.131
Face with mouth motion														
Eye	4.79	.029	0.00	.985	***8.09***	< .001*	2.94	.087	***9.43***	< .001*	0.60	.549	***8.83***	< .001*
Mouth	0.24	.626	0.01	.931	4.26	.015	1.77	.184	4.31	.014	0.01	.989	3.57	.029
Biological motion														
Upright	0.25	.616	0.11	.746	1.51	.221	0.24	.622	0.62	.539	0.34	.715	0.90	.406
Inverted	7.51	.006	0.00	.999	1.45	.237	0.08	.774	1.06	.347	0.28	.757	0.98	.377
The preference paradigm														
People	***8.36***	.004*	***10.39***	.001*	3.73	.025	0.43	.511	1.88	.154	0.02	.982	0.22	.802
Geometry	4.31	.039	***10.59***	.001*	***6.09***	.003*	2.18	.140	1.17	.311	0.90	.407	0.36	.700
Finger-pointing														
Social	3.09	.080	0.65	.420	1.08	.342	0.63	.429	0.19	.830	0.29	.752	0.02	.976
Geometry	***14.96***	< .001*	0.00	.957	0.66	.517	2.06	.152	0.32	.725	1.04	.355	1.36	.258

*p < .005 (.05/10)

### Face with mouth motion

**Fig. 3:**
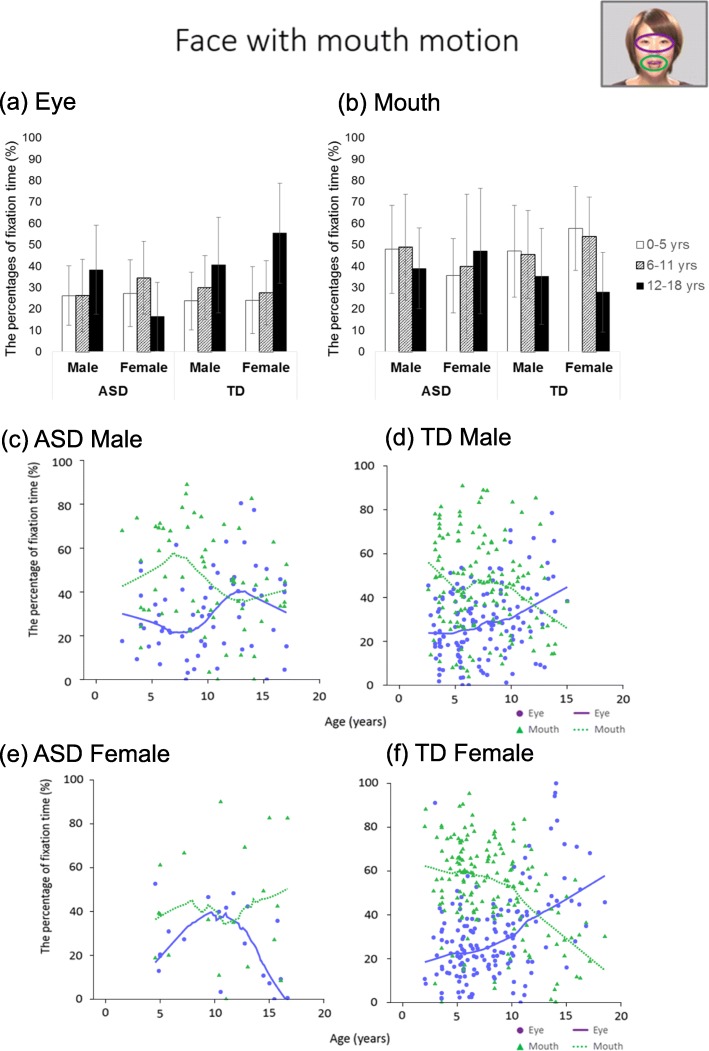
Bar graphs and LOESS curves for “face with mouth motion.” **a**, **b** The bar graphs of the ASD group and TD group for the percentage fixation times of the eye and mouth regions, respectively. Error bars indicate standard errors of the mean. **c**–**f** Scatter plots and LOESS curves of the ASD group and TD group, respectively. Purple circles and lines show data from the eye region, and yellow green triangles and dashed lines show data from the mouth region

For the eye region, the analysis revealed a three-way interaction (*F* (2, 378) = 8.83, *p* < .001, *η*^2^ = 0.045). Post hoc comparisons showed that the male participants in the TD 12–18-year group gazed significantly longer at the eye region than did the TD male 0–5-year group (*p* = .001), and the female participants in the TD 12–18-year group gazed significantly longer at the eye region than did the participants in 0–5 and 6–11-year groups (ps < .001). Conversely, in both ASD sex groups, there were no age-group differences (ps > .050). Furthermore, there were sex differences only in the 12–18-year group for both the ASD and TD groups, while the TD female group gazed significantly longer at the eye region than did the TD male group (*p* = .006); the ASD female group gazed significantly less at the eye region than did the ASD male group (*p* = .003). As a significant sex difference was found on the ANOVA, we present separate LOESS plots for each sex in Fig. 3. Especially remarkable in girls, the regression line in both ASD groups showed a sharp decline of attention to the eye region after around 10 years of age. Conversely, there was a constant rise of attention to the eye region in the TD group.

There were no main effects and interactions for the mouth region (Fs ≤ 4.31, ps ≥ .014). As is the case with the face without mouth motion, the LOESS plot in Fig. 3 shows that there was a rise in attention to the mouth region in the ASD group after around 10 years of age, whereas there was a constant decline in attention to the mouth region in the TD group.

These results are shown in Table 2 and Fig. 3.

### Biological motion

**Fig. 4:**
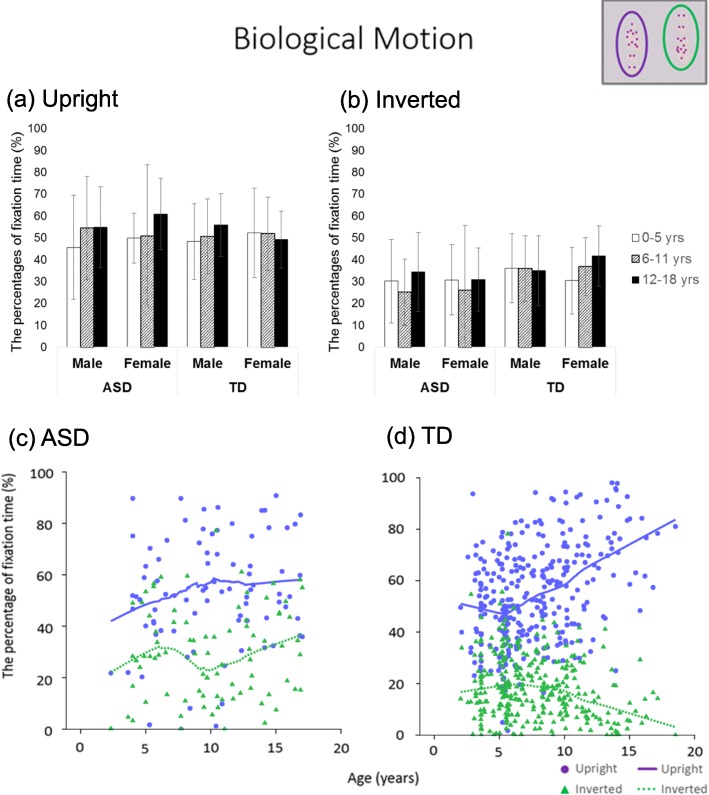
Bar graphs and LOESS curves for “biological motion.” **a**, **b** The bar graphs of the ASD group and TD group for the percentage fixation times of upright and inverted biological motion, respectively. Error bars indicate standard errors of the mean. **c**, **d** Scatter plots and LOESS curves of the ASD group and TD group, respectively. Purple circles and lines show data from upright biological motion, and yellow green triangles and dashed lines show data from inverted biological motion

There were no main effects and interactions both in upright and inverted biological motion (Fs ≦ 7.51, ps ≧ .006) (Table 2 and Fig. 4). Both the ASD and TD groups did not show a sharp rise or decline as shown in the LOESS plot in Fig. 4.

### The preference paradigm

For the people region, the main effect of diagnosis was also significant (*F* (1, 378) = 8.36, *p* = .004, *η*^2^ = 0.022), and the TD group gazed longer at the people region than did the ASD group. In addition, there was a significant main effect of sex (*F* (1, 378) = 10.39, *p* = .001, *η*^2^ = 0.027), and the female group gazed longer at the people region than did the male group. The LOESS plot indicated that the percentage of fixation to the people region gradually decreased in the TD group and stabilized after around 5 years of age. The regression line for the ASD group declined until 10 years of age and showed a limited rise after this age.

The main effect of sex was also significant for the geometry region (*F* (1, 378) = 10.59, *p* = .001, *η*^2^ = 0.27), and the male group gazed longer at the geometry region than did the female group. Furthermore, the main effect of age was significant (*F* (1, 378) = 6.09, *p* = .003, *η*^2^ = 0.31). Post hoc comparisons showed that the 0–5-year group gazed less at the geometry region than did the other two age groups (ps < .001). The regression lines of both the ASD and TD groups showed the opposite trend to that for the people region.

**Fig. 5:**
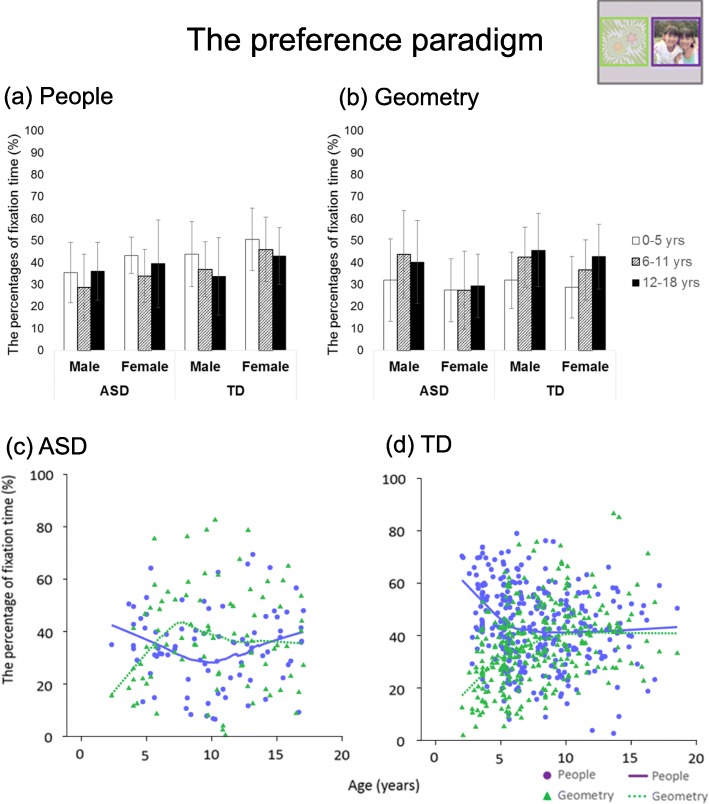
Bar graphs and LOESS curves for the preference paradigm. **a**, **b** The bar graphs of the ASD group and TD group for the percentage fixation times of the people and geometry regions, respectively. Error bars indicate standard errors of the mean. **c**, **d** Scatter plots and LOESS curves of the ASD group and TD group, respectively. Purple circles and lines show data from the people region, and yellow green triangles and dashed lines show data from the geometry region

These results are shown in Table 2 and Fig. 5.

### Finger-pointing

**Fig. 6:**
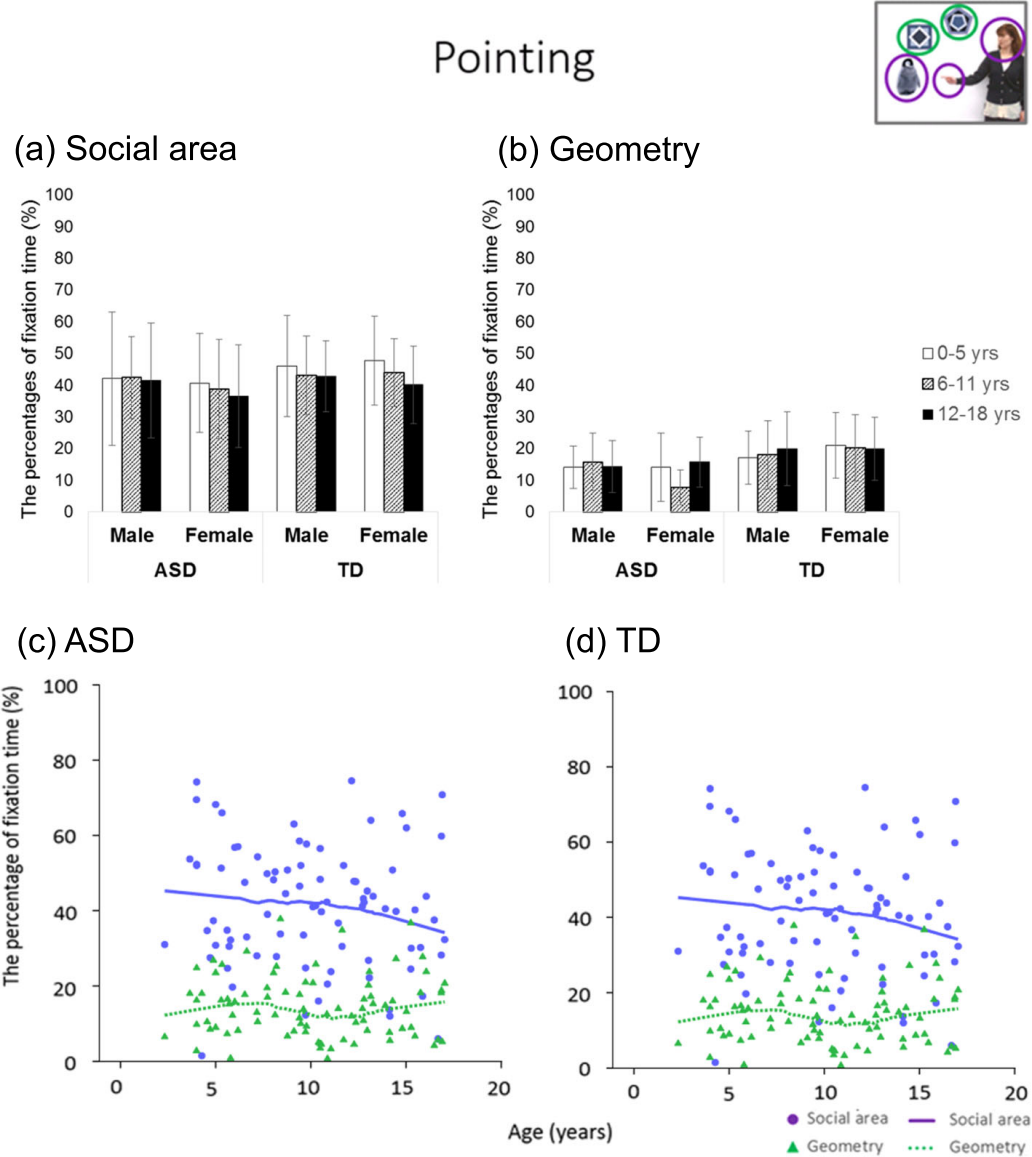
Bar graphs and LOESS curves for “finger pointing.” **a**, **b** The bar graphs of the ASD group and TD group for the percentage fixation times of objects with or without finger pointing, respectively. Error bars indicate standard errors of the mean. **c**, **d** Scatter plots and LOESS curves of the ASD group and TD group, respectively. Purple circles and lines show data from objects with finger-pointing, and yellow green triangles and dashed lines show data from objects without finger-pointing

For the social region, there were no significant main effects or interactions (Fs ≤ 3.09, ps ≥ .080). For the geometry region, the main effect of diagnosis was significant (*F* (1, 378) = 14.96, *p* < .001, *η*^2^ = 0.038), and the TD group gazed longer at the geometry region than did the ASD group. Both the ASD and TD groups did not show a sharp rise or decline in the LOESS plot. These results are shown in Table 2 and Fig. 6.

### Correlation analysis

As there were main effects and interactions of the age group and sex in the face stimuli and the preference paradigm, we carried out partial correlation analysis using age and sex as covariates. At first, we carried out a correlation analysis between the SRS-2 scores and the percentage fixation time to AoIs using age and sex as covariates. In the whole group analysis, we found that the SRS-2 total score was significantly negatively correlated with three items: inverted biological motion in biological motion in the 0–18-year group and the people region in the preference paradigm in both the 0–18-year and 6–11-year groups. In addition, the percentage of fixation to the geometry region in finger-pointing showed a significant positive correlation with the SRS-2 total score in the ASD 0–18-year group. The other correlation coefficients were not significant, and all results are shown in Table 3. Next, we carried out a correlation analysis between the IQ/DQ scores and the percentage fixation time to AoIs in the ASD group using age and sex as covariates. There were no significant correlations, and all results are shown in Table 4.

**Table 3:** Results of partial correlation analysis between SRS-2 scores and the percentage fixation time to AoIs using age and sex as covariates

		0–18 years		0–5 years		6–11 years		12–18 years	
		*r*	*p*	*r*	*p*	*r*	*p*	*r*	*p*
Whole group									
Face without mouth motion	Eye	− .114	.024	.004	.961	− .044	.555	− .270	.030
	Mouth	.095	.062	− .059	.494	.033	.656	.328	.008
Face without mouth motion	Eye	− .127	.012	.007	.940	− .079	.287	− .261	.036
	Mouth	.033	.514	− .014	.870	.016	.833	.127	.314
Biological motion	Upright	.048	.347	− .061	.477	.069	.354	.094	.456
	Inverted	− **.150**	.003*	− .007	.937	− **.212**	.004*	− .147	.243
The preference paradigm	People	− .114	.025	− .107	.215	− **.235**	.001*	.018	.889
	Geometry	− .101	.048	− .065	.451	.021	.779	− .284	.022
Finger-pointing	Social	− .025	.620	− .055	.522	− .040	.593	− .018	.885
	Geometry	− .080	.115	− .074	.392	− .046	.541	− .190	.129
ASD group									
Face without mouth motion	Eye	− .082	.464	− .028	.916	.019	.918	− .236	.227
	Mouth	.165	.141	.319	.213	.020	.912	.240	.218
Face without mouth motion	Eye	− .114	.310	− .097	.710	− .112	.540	− .084	.671
	Mouth	− .012	.916	.340	.182	− .181	.323	− .141	.475
Biological motion	Upright	.072	.525	.183	.482	− .012	.948	.146	.459
	Inverted	− .057	.616	− .212	.415	.129	.483	− .241	.217
The preference paradigm	People	− .051	.648	.403	.108	− .226	.213	.010	.960
	Geometry	.051	.651	.047	.858	.126	.491	− .186	.343
Finger-pointing	Social	.035	.755	.258	.316	− .177	.333	.041	.837
	Geometry	**.313**	.004*	.602	.011	.357	.045	.127	.518
TD group									
Face without mouth motion	Eye	.022	.705	− .020	.828	.046	.583	.169	.331
	Mouth	− .028	.630	− .035	.704	.010	.904	− .113	.520
Face without mouth motion	Eye	− .007	.898	− .068	.467	− .025	.763	.124	.479
	Mouth	.018	.758	.076	.415	.076	.361	− .200	.249
Biological motion	Upright	− .029	.616	− .095	.304	.091	.269	− .271	.115
	Inverted	.045	.438	.161	.081	-.083	.318	.272	.115
The preference paradigm	People	.000	.995	− .020	.829	− .016	.851	.126	.472
	Geometry	− .037	.517	− .068	.462	.046	.581	− .212	.221
Finger-pointing	Social	.030	.598	− .037	.689	.060	.466	.192	.270
	Geometry	.037	.525	.003	.978	.102	.218	− .132	.450

*p < .005

**Table 4:** Results of partial correlation analysis between IQ/DQ scores and the percentage fixation time to AoIs using age and sex as covariates in ASD group

ASD group		0–18 years		0–5 years		6–11 years		12–18 years	
		*r*	*p*	*r*	*p*	*r*	*p*	*r*	*p*
Face without mouth motion	Eye	− .008	.941	− .096	.715	− .061	.742	.133	.501
	Mouth	.060	.596	.135	.605	.119	.515	− .076	.700
Face without mouth motion	Eye	− .006	.954	− .317	.215	− .023	.899	.127	.518
	Mouth	− .065	.565	.028	.915	− .083	.652	− .021	.915
Biological motion	Upright	.001	.991	− .101	.701	− .042	.818	.161	.414
	Inverted	.059	.602	− .077	.768	.288	.109	− .161	.413
The preference paradigm	People	.037	.742	− .204	.431	− .056	.761	.268	.169
	Geometry	− .071	.526	− .180	.490	.145	.429	− .338	.079
Finger-pointing	Social	− .154	.169	.036	.892	− .152	.407	− .278	.151
	Geometry	.120	.284	− .007	.980	**.363**	.041	− .089	.652

*p < .005

## Discussion

The aim of this study was to identify the developmental changes in gazing at social information in individuals with ASD and TD individuals from childhood to adolescence. In the face stimuli, the ANOVA results showed that the ratio of gazing at the eye region rose with increasing age in the TD group. When being shown videos of faces “without mouth motion,” the TD group showed reductions in the LOESS curves for the ratio of gazing at the eye region, along with increasing age until around 5 years of age and later showed a tendency for the curves to rise with increasing age. Conversely, in the ASD group, the LOESS curves of the ratio of gazing at the eye region continued to rise until around age 10 years and showed a tendency to decrease thereafter; a pattern especially pronounced in girls with ASD. The main effect of diagnosis was significant in the gaze ratio at the people region in the preference paradigm, with the ASD group’s gaze ratio being lower than that of the TD group’s.

In the ASD group, the ANOVA results for “face with mouth motion” did not show any increase in the gaze ratio at the eye region in line with increasing age as seen in the TD group. Especially, in female participants with ASD in “face with mouth motion,” the LOESS regression curve in both face stimuli showed reduction in gazing at the eye region and increase in gazing at the mouth region at around age 10 years, although it is necessary to emphasize that ANOVA does not suffice to examine significant developmental changes. While the previous study that attempted to clarify the developmental changes in attention to social information in ASD targeted early childhood [37], we evaluated developmental changes in children throughout childhood and adolescence and delineated the developmental trajectories of complex behaviors. Our findings could be instrumental in elucidating the developmental and clinical characteristics of ASD. Another previous study reported on the developmental trajectories of social interaction from diagnosis through age 14 years, and these trajectories appeared to decline at ages greater than 10 years [48]. This clinical picture may be related to the reduction in gazing at the eye region and increase in gazing at the mouth region at around age 10 years that was observed in this study, and there may be qualitative differences in abnormal eye contact in ASD between individuals in early childhood and those older than 10 years.

Why, then, does the ratio of gazing at the eye region decrease in individuals with ASD older than 10 years as shown in the LOESS regression curve in both face stimuli? Some hypotheses are available to explain the lower attention to social information in individuals with ASD: the social motivation hypothesis [49], social aversion hypothesis [14, 50, 51], and “dynamics of a clinical phenotype” hypothesis [52, 53]. Social motivation refers to the mechanism by which people turn their attention to social stimuli, seek pleasure in social interaction and try to perceive it, and foster and maintain social connections [49]. According to the social motivation hypothesis, people with ASD have characteristically low social motivation, and because of this, their focus on social information is weak. In fact, the orbitofrontal–striatal–amygdala circuit, which responds to social stimuli such as faces and social approval, has been repeatedly highlighted as abnormal in ASD [49]. The social aversion hypothesis contests that individuals with autism avoid gazing at social information as a result of the considerable resources required to process such social information, especially that of the eye region in people’s faces. One related factor is likely to be anxiety, which is reported to reduce the gaze ratio at the eye region [54, 55]. Reportedly, the amygdala, which is a rapid detector of aversive environmental stimuli and gives rise to affective or behavioral states to allow for adaptive responses to potential threats [56], becomes strongly activated as a result of gazing at the eye region in individuals with anxiety disorder [57] and individuals with ASD [51, 58]. This is probably why such individuals avoid looking other people in the eyes. Anxiety disorder is highly comorbid with ASD [59–61], with the onset of social anxiety disorders, in particular, reported to peak between the ages of 10 and 15 years [62]. Therefore, individuals with ASD after the age of 10 years showed an aversion to the eye region, which is typically processed as a reward in TD individuals [63], due to high anxiety. The “dynamics of a clinical phenotype” hypothesis states: “early emerging behavioral symptoms alter the child’s self-directed patterns of attention, changing their experience of the environment and further restricting social learning opportunities” [52]. Thus, it is claimed that social behaviors including eye contact modulate cortical activation even in infants and that social experience may affect social behavior itself [52, 53]. Because this was a cross-sectional study, we cannot deny the possibility that the older participants with ASD may have acquired the gaze behaviors described above through previous experience. The present findings do not provide evidence for any of these hypotheses; therefore, it is necessary to confirm them in the future.

Main-effects of diagnosis were shown for the people region in the preference paradigm and for the geometry region in the finger-pointing movies. Interestingly, although the people (social) region and the geometry region were set as AoIs in both stimuli, the participants in the ASD group gazed more briefly at the people region in the preference paradigm and the geometry region in the finger-pointing movie. These ambivalent results are considered to depend on the quality of the stimuli. Both the people and geometry regions moved in the preference paradigm, and only the social region moved in the finger-pointing movie. A previous study reported that individuals with ASD very attentively watched the hands that moved in the finger-pointing video [7]. Therefore, the possibility can also be considered that because the subjects were gazing attentively at the moving hands, there were no differences between the ASD and TD groups. Even if a researcher determines similar outcomes, opposite results may be obtained depending on the quality of the stimuli. Gazing behavior is very delicate, and researchers should be able to use the same stimuli across studies to enable comparisons.

For the preference paradigm, the ASD group reportedly gazed more briefly at the people region than did the TD group in previous studies targeting both children [26–30] and adults [22]. All the aforementioned studies with children [26–30] targeted early childhood, and to the best of our knowledge, our study was the first study to report on children during the course of childhood and adolescence. Considering our results, we contest that, regardless of age, the ratio of gazing at the people region in the preference paradigm may reflect the characteristic of sociability in ASD.

Regarding geometric shapes, the main effect of age group was significant. Although this was seen especially conspicuously in TD individuals, the participants gazed at geometric shapes longer as they grew older. This may imply that, along with increased age, individuals become more able to “search” for stimuli other than social stimuli. In other words, although individuals appear to look at social stimuli automatically [64–66], they may become able to divert their attention to non-social stimuli with increasing age.

Regarding the perception of biological motion under passive view conditions targeting children, the findings of previous studies are inconsistent, with some stating that children with ASD gaze less at biological motion [31–33], while others contesting that they gaze more [37]. Studies targeting adults found no significant difference between ASD and TD groups [22]. Studies conducted by Fujisawa et al. [37] and Fujioka et al*.* [22] found that individuals with ASD gazed at biological motion approximately the same as, or more than, TD individuals, using Gazefinder, as was used in our study [22, 37]. Therefore, the quality of the stimuli may also be related. A previous study reported that, if a nonsocial sound is attached to biological motion (response to nonsocial-physical contingencies that are disregarded by control children), or, in other words, if a clapping sound is simultaneously presented with the biological motion of hand clapping, children with ASD tend to look at the biological motion very attentively [31]. Biological motion in Gazefinder shows people dancing to a song, indicating a high rate of synchronization between sound and movement. Differentiation between ASD and TD may therefore be difficult with stimulation by Gazefinder.

For the partial correlation analysis using age and sex as covariates, there were a few significant correlations between the SRS-2 total score and the percentage fixation time to AoIs. Previous research with adults with ASD using Gazefinder also reported this trend and concluded that the SRS-2 can measure a wide range of social deficits, whereas Gazefinder can only measure abnormality in eye contact, which is only one social deficit component [22]. However, some studies have reported significant correlations between attention to social information and the SRS-2 score [16] and other studies have reported significant correlations between attention to social information and the scores on other instruments, such as the Autism Diagnostic Observation Schedule (ADOS) [15, 17], while other studies have shown no significant correlations between them [16, 23]. It will be necessary to clarify the types of abilities that are associated with attention to social information in the future, although it may be affected by the quality of the stimuli and the age of the participants. Furthermore, there were no significant correlations between the IQ/DQ scores and the percentage fixation time to AoIs in the ASD group. Based on these results, attention to social information and IQ/DQ scores may be independent, at least in individuals with ASD.

This study made an important discovery regarding developmental changes in gazing at social information in the TD group. In TD group for “a face without mouth motion,” the ANOVA and LOESS results showed that the gaze ratio at the eye region decreased and the ratio at the mouth region increased up to around 5 years of age, and afterward, the opposite trends were obtained. Considering previous findings that TD children gradually gaze more briefly at strong social information along with increasing age [37, 39, 67] and that TD adults tended to gaze longer at the eye region than did TD children [11, 18], there is a discrepancy in that gazing at areas carrying strong social information decreases or does not change along with increasing age in childhood, whereas adults often gaze longer at such information than do children. The results of our study regarding faces without mouth motion explain this discrepancy. Then, why does the gaze ratio at the eye region decrease and the gaze ratio at the mouth region increase up to approximately 5 years of age in faces without mouth motion? A previous study reported that the fixation time to the mouth region at 0.5 years (6 months) old predicted expressive language at 2 years old [68], and participants up to 5 years who show significant language development may gaze longer at the mouth region at approximately 5 years old even in the faces without mouth motion. Subsequently, after 5 years of age, TD individuals could be able to divert their attention from the mouth and pay more attention to the eye region. Besides, in “face with mouth motion,” the moving mouth was regarded to have attracted the attention of young children, and, as a result, gazing at the eye region showed an increase only in conjunction with age in this study. As such unique developmental changes in attention to social information cannot be confirmed with other stimuli, the eye region may hold a special place among the social information-carrying stimuli.

## Limitations

We cannot deny the possibility that, because this was a cross-sectional study, the degree of severity and the degree of social interest might have differed according to the subjects’ age group. In addition, in the ANOVA using age as a dependent variable for age group, there were group differences for age, sex, and/or diagnosis, in other words, there were differences in age among the sub-groups. These sub-group differences in age do not affect the interpretation of LOESS; however, they may have affected the ANOVA results when using the percentage fixation time to AoIs as the dependent variable. We believe that we will be able to further validate the findings of this study by clarifying the developmental changes in gazing at social information with longitudinal studies strictly controlling for the severity of ASD and age. In addition, especially in the ASD group, the sample size in each age group was somewhat small. On ANOVA analysis, the *p* value of some items was below .05, which is a commonly used significance level, but not below .005, which was the significance level we employed in this study adapted from Bonferroni corrections. Moreover, the sample size of the 0–5-year ASD group was considerably smaller than that of the other ASD age groups, as was the sample size of the 12–18-year TD group compared to the other TD age groups. As a result, it was challenging to obtain significant results with ANOVA, and the LOESS outcome may have been distorted. Although we revealed valuable findings in this study, it may be possible to reveal new trends by performing analysis with a larger and homogeneous group. In this study, we did not use gold-standard evaluations for diagnosis such as the ADOS and Autism Diagnostic Interview-Revised (ADI-R). As a previous study reported that the clinical diagnosis in the 2nd year of life without using the ADOS or ADI-R was stable [69] and the participants with ASD in this study were diagnosed by certified psychiatrists of the Japanese Board of Psychiatry or board-certified pediatricians of the Japan Pediatric Society, we are quite confident that the possibility of extreme misdiagnosis is very limited. However, from the viewpoint of group homogeneity and research, it would be preferable to evaluate participants using such tools in the future. In addition, a major limitation of this study is that no evaluation tools were used except for the SRS-2, and the IQ in the TD group was not measured. Therefore, the present results may have been affected by various factors. Future studies will be needed to measure and control for characteristics that affect attention to social information such as anxiety. All participants in this study were Japanese, and previous studies with Japanese and British adults/children have reported that Japanese participants looked longer in their eye area than did British participants [70, 71]. Confirmation is needed that our results can be replicated across ethnicities and cultures.

## Conclusions

The purpose of this study was to identify, in children with ASD and TD children, the developmental changes in gazing at social information from childhood to adolescence. “Face without mouth motion” revealed characteristic developmental changes in the TD group, in which the gaze ratio at the eye region decreased up to approximately age 5 years but increased thereafter. The ASD group showed a reduction in gazing at the eye region around the age of 10 years. These results suggest that the eye region constitutes a special stimulus among the stimuli carrying social information. The main effect of diagnosis was significant in the gaze ratio at the people region in the preference paradigm, with the gaze ratio in the ASD group being lower than that in the TD group. The people region in the preference paradigm may be a stimulus that reflects the sociability of ASD individuals regardless of age. These developmental changes in the gaze ratio appear to be a major characteristic of ASD and were first clarified in our study that investigated developmental changes using a broader age group of 3 to 18 years. However, factors that could cause such developmental changes could not be identified in this study and warrant further research. Furthermore, if these factors are related to secondary disorders, as research progresses, it may be possible to predict secondary disorders and determine the methods and timing of intervention for these disorders by longitudinally measuring attention to social information.

## Data Availability

The datasets generated and/or analyzed during the current study are not publicly available because they were involved in a clinical trial but are available from the corresponding author on reasonable request.

## Abbreviations

ADI-R: Autism Diagnostic Interview-Revised
ADOS: Autism Diagnostic Observation Schedule
AoI: Area-of-interest
ANOVA: Analysis of variance
ASD: Autism spectrum disorder
DQ: Developmental quotient
IQ: Intelligent quotient
LOESS: Locally weighted scatterplot smoothing
SRS-2: Social Responsiveness Scale Second Edition
TD: Typically developed
WISC-IV: Wechsler Intelligence Scale for Children-Fourth Edition
